# Conceptualization of an Ex Vivo Organ Culture (EVOC) Model for Human Seminoma: A Pilot Study

**DOI:** 10.3390/ijms27010452

**Published:** 2025-12-31

**Authors:** Grigory Demyashkin, Vladimir Shchekin, Mikhail Parshenkov, Petr Shegay, Andrei Kaprin

**Affiliations:** 1 Department of Digital Oncomorphology, National Medical Research Centre of Radiology, 2nd Botkinsky Pass., 3, 125284 Moscow, Russia; 2 Laboratory of Histology and Immunohistochemistry, Institute of Translational Medicine and Biotechnology, I.M. Sechenov First Moscow State Medical University (Sechenov University), Trubetskaya St., 8/2, 119048 Moscow, Russia; 3 Department of Urology and Operative Nephrology, Peoples’ Friendship University of Russia (RUDN University), Miklukho-Maklaya Str. 6, 117198 Moscow, Russia

**Keywords:** ex vivo organ culture, EVOC, seminoma, germ cell tumor, Ki-67, tumor microenvironment

## Abstract

Personalized treatment strategies for seminoma, a germ cell tumor, are crucial due to inherent tumor heterogeneity. Existing in vitro models often inadequately represent the native tumor microenvironment. Ex vivo organ culture (EVOC) offers a potential solution by preserving the tumor’s original architecture and cellular interactions. This study presents the pilot study for adaptation of the EVOC platform specifically for non-metastatic seminoma, focusing on short-term tissue maintenance and an assessment of viability markers, examining intraoperative biopsies from 12 patients with non-metastatic seminoma (cT1–2, cN0–3, M0) undergoing orchifuniculectomy. Tissues were cultured in DMEM/F12 medium supplemented with fetal bovine serum and antibiotics. Histological and immunohistochemical analyses were performed on days 0, 3, 7, and 10. We analyzed the proliferative index (PI), using Ki-67; total cell number (OCN); and tumor cell number (TCN; PLAP-positive cells). The area under the curve (AUC) for PI was calculated to evaluate tumor viability. Statistical analyses included repeated measures ANOVA and post-hoc tests. Histological examination confirmed the preservation of the native seminoma histoarchitecture up to day 7. OCN showed a median decrease of 32.6% on day 7 (*p* = 0.002) and 55.1% on day 10 (*p* = 0.0004) compared with the baseline. TCN showed a median decrease of 27.5% on day 7 (*p* = 0.0033) and 53.2% on day 10 (*p* = 0.00018) compared with the baseline. The PI decreased significantly from day 3 to day 10 (*p* < 0.05). The AUC for PI was identified as a representative marker of tumor viability. An “EVOC score” calculation method was proposed to compare the effects of different treatments. This proof-of-concept work confirms that seminoma tissue can be maintained ex vivo for up to ten days under optimized conditions. The EVOC system developed here will serve as a methodological basis for further improving culture stability and exploring its broader applications in tumor biology and pharmacological testing.

## 1. Introduction

Cancer encompasses a heterogeneous group of oncological diseases, distinguished not only by the tissue origin and morphological characteristics but also by molecular and biological parameters, even within the same histological subtype of tumor. This poses a significant obstacle to selecting an adequate anti-cancer therapy regimen [1,2]. Another challenge for oncologists in choosing treatment strategies for patients with malignant neoplasms is tumors resistant to standard anti-cancer therapy protocols, which prompts researchers to seek methods for selecting drugs and other targeted therapies based on developments in tissue, cellular, and molecular biology [3].

Nowadays, anti-cancer therapies should consider the histological type and biology of the tumor, that is, the interaction of atypical cells and elements of the tumor microenvironment, the properties of which can influence, among other things, the tumor’s resistance to certain drugs [4,5,6]. Moreover, many currently developed drugs target not so much the tumor cells themselves as the elements of the tissue landscape. For example, targeted drugs that inactivate the PD-L1 ligand, expressed by tumor cells, thereby preventing immune evasion, are a colossal success of modern oncology [7,8]. Drugs targeting CTLA-4 have a similar mechanism of action [9]. Drugs aimed at modulating the vascular component of the tumor, suppressing neoangiogenesis—antibodies to vascular endothelial growth factor (VEGF)—are also actively used in routine practice. This is especially important, as the impairment of blood supply to tumor cells naturally leads to a limitation of their proliferative potential and death [10,11].

**Figure 1:**
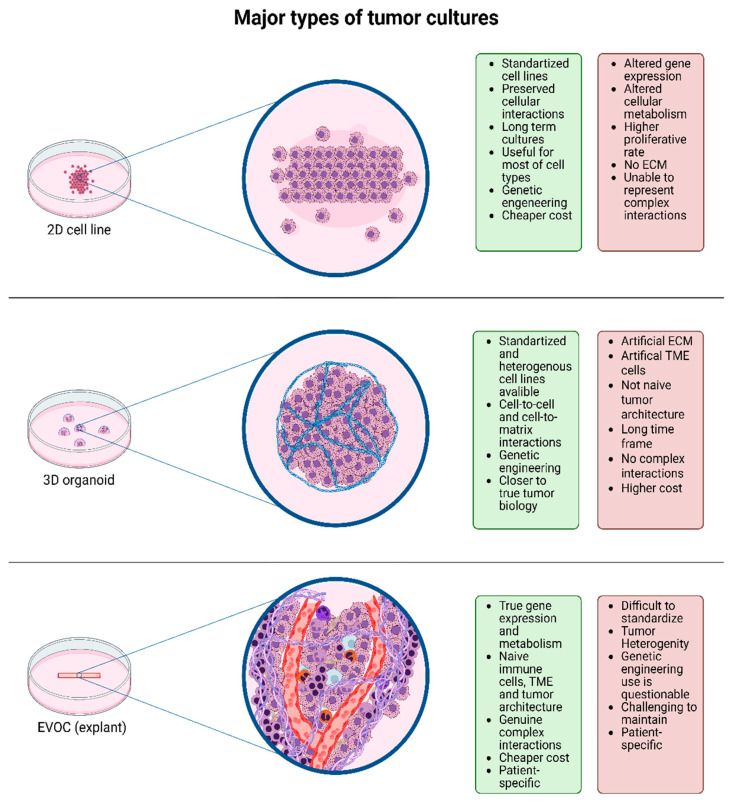
Main models of cell/tissue cultures. Abbreviations: ECM—extracellular matrix; TME—tumor microenvironment, EVOC—ex vivo organ culture.

However, most of the cell and molecular biology methods used are limited in their ability to recreate the native structure of both the tumor itself and the tumor microenvironment (Figure 1). Thus, two-dimensional (2D) cell cultures, despite representing most of the biological characteristics of atypical cells, do not provide the opportunity to study their microenvironment and true intercellular interactions between tumor cells, which is sometimes reflected in the distortion of, for example, the immunohistochemical profile of cells compared to primary tumors [12].

Three-dimensional (3D) cell structures and organoids have become a significant step in studying the biological characteristics of tumors, allowing the recreation of a conformation of intercellular interactions of atypical cells that is close to native. However, they are still not able to adequately represent the native structure of the tumor due to the technological features of organoid creation, including cell extraction, treatment with proteolytic enzymes, and the introduction of artificial components of the extracellular matrix [13,14].

Ex vivo organ culture (EVOC), or explant technology, has recently regained attention as a promising experimental platform. EVOC is experiencing a kind of renaissance [15], as it has advantages compared to the above-described methods of studying tumors. Explants are tissue fragments extracted from a primary tumor and placed in a special nutrient medium. That is, the key advantages in this case are the preservation of the intercellular interactions and microenvironment structure as close as possible to the original [16]. EVOC technology offers a potential platform for personalized medicine (taking into account future research and extensive cross-functional work by researchers in related fields) in various areas of oncology, and its application to germ cell tumors, particularly seminomas, opens up opportunities for studying fundamental aspects of tumor biology.

Unlike many epithelial cancers, seminoma inherently embodies a unique biological complexity, characterized by the co-existence of germ and somatic cell lineages, an intricate vascular network, and a dynamic stromal and endocrine microenvironment [17]. This multifaceted composition, which can be reproduced in EVOC models, offers a critical advantage over conventional 2D/3D cell cultures and even organoids, which often fail to recapitulate the full spectrum of intercellular interactions and microenvironmental cues crucial for understanding tumor progression and resistance. Furthermore, while patient-derived xenografts provide invaluable in vivo insights, their high cost, extended timelines, and potential for species-specific immunological discrepancies underscore the immediate and translational utility of EVOC [18]. Therefore, our focus on seminoma within an EVOC framework is not merely a methodological choice but our deliberate strategic imperative, enabling an unprecedented, high-fidelity investigation into a tumor type whose developmental origins and complex microenvironment demand a model that transcends the limitations of existing platforms, thereby paving the way for novel therapeutic strategies and a deeper understanding of germ cell tumorigenesis.

Seminoma represents a subtype of germ cell tumors with distinctive developmental and biological features. The formation of germ cell tumors is a multi-stage process that begins during embryogenesis, when one or another damaging event leads to the arrest or aberrant differentiation of primordial gonocytes [19,20]. Subsequently, these atypical cells remain dormant until puberty, when exposure to sex hormones stimulates their further proliferation and differentiation, accompanied by the accumulation of an even greater number of genetic abnormalities, which, coupled with the influence of environmental factors, lead to the final malignant transformation of these cells [21]. Also, seminoma is characterized by a macrophage-rich tumor microenvironment, the formation of which occurs, among other things, due to the active secretion of prostaglandins, which attract immune cells [22,23,24]. This immune- and macrophage-rich milieu may influence tumor behavior and therapeutic responses [25], underscoring the need for models that preserve the native stromal–immune architecture. However, existing in vitro systems fail to preserve this complex tissue architecture and stromal–immune context, making them unsuitable for the accurate modeling of seminoma biology and drug sensitivity.

An important yet insufficiently explored aspect of EVOC systems is the temporal window during which tumor tissue can retain structural integrity and biological activity ex vivo. Most published EVOC studies of seminoma and related germ cell tumors have focused on short-term cultures, typically limited to approximately 5–7 days, largely due to concerns regarding progressive tissue degeneration and loss of proliferative capacity [26,27]. However, evidence from organ culture models of other testicular tissues suggests that, under optimized conditions, the preservation of tissue viability beyond one week may be achievable. Defining the upper temporal limits of seminoma viability ex vivo is therefore critical for expanding the experimental and translational utility of EVOC platforms, particularly for applications requiring extended observation periods, as dynamic proliferation assessments or delayed treatment responses.

This gap emphasizes the need for a model that maintains the native tumor organization and microenvironment. In this context, we present our pilot study on adapting an ex vivo organ culture platform specifically for seminoma. We hypothesize that adapting the EVOC platform for seminoma will enable the prolonged preservation of tissue integrity and cell viability, allowing a quantitative assessment of proliferative potential.

### Research Objective

Aim of the study: to establish an optimized ex vivo organ culture (EVOC) model specifically for non-metastatic seminoma, with a primary focus on assessing the sustained viability of atypical cells through the quantitative evaluation of the Ki-67 proliferation index within its native tissue context.

## 2. Results

### 2.1. Histological Examination

**Figure 2:**
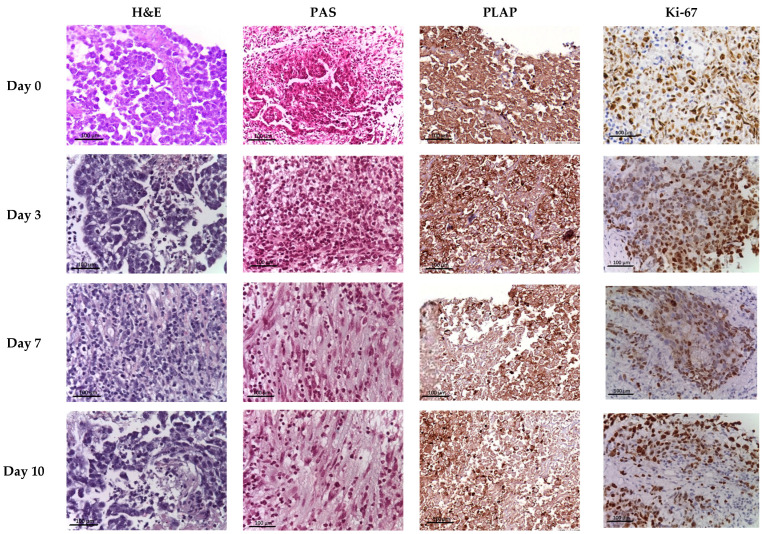
Seminoma EVOC based on all time-points. Staining with Hematoxylin and eosin and PAS; immunohistochemical reactions with antibodies to PLAP and Ki-67; magnific., ×250.

In the testicular samples of patients (n = 12; 100%) at day 0 (D0, EVOC), seminoma, a subtype of germ cell tumor (GCT), with a lobular structure was detected. Adjacent non-tumorous testicular tissue frequently exhibited total germinal aplasia, a common finding in association with testicular germ cell tumors, suggesting a potential underlying testicular dysfunction or predisposition. Most atypical cells had light cytoplasm and clear borders, with polygonal nuclei containing one or more nucleoli. The stromal component was represented by fibrous connective tissue forming septa. Moderate cellular inflammatory infiltration, characterized by the accumulation of lymphocytes, plasma cells, and other immunocompetent cells, was found within the tumor and paratumoral tissue. Focal intercellular edema, microcysts, and small foci of coagulation necrosis were occasionally observed, particularly within the tunica albuginea, where inflammatory infiltration was also present. These degenerative changes were generally limited and did not compromise the overall tissue integrity in the main tumor mass, as confirmed through subsequent viability assessments. In the microsections of the surgical material, the morphological picture of the seminoma pT1 stage was visualized: the tumor was limited to the testis and its rete without vascular or lymphatic invasion. Tumor infiltration patterns varied among patients: in eight patients (72.2%), infiltration was confined to the tunica albuginea but did not extend to the tunica vaginalis. In the remaining four patients (27.8%), seminoma exhibited vascular or lymphatic invasion and infiltration into both the tunica albuginea and tunica vaginalis of the testis (Figure 2).

### 2.2. Histochemical Examination

Upon Periodic Acid–Schiff (PAS) staining, atypical cells exhibited pronounced eosinophilic staining of the cytoplasm, indicating the consistently abundant accumulation of intracellular glycogen, a characteristic metabolic feature of atypical cells (Figure 2).

### 2.3. Immunohistochemical Examination

In the immunohistochemical analysis, PLAP-positive atypical cells were detected in all cases (n = 12) throughout the observation period, but their numbers decreased (Table 1; Figure 2).

**Figure 3:**
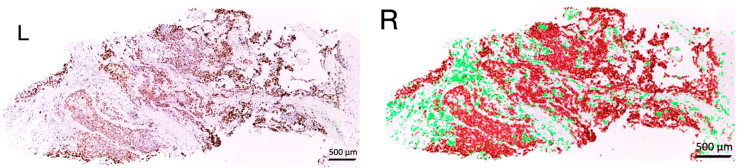
Seminoma’s map. Immunohistochemical study with antibodies to Ki-67. **Left** (L)—histoscan. **Right** (R)—dot plot showing distribution of Ki-67-positive tumor cells in histoscan. (red dots—Ki-67-positive cells, green—Ki-67-negative cells); magnific., ×20.

In the immunohistochemical analysis with antibodies to Ki-67, the number of actively proliferating atypical cells (PI of seminoma) decreased as early as day 3. The results of immunohistochemical studies and their interpretation are detailed below (Figure 2 and Figure 3).

### 2.4. EVOC Model Morphometry

By quantifying the overall cell number (OCN) in seminoma EVOC samples, no statistically significant difference was observed between day 0 and day 3 (*p* = 0.071), indicating the short-term preservation of total cellularity. In contrast, a statistical reduction in OCN was detected at later time points, with a median decrease of 32.6% (interquartile range [IQR]: 28.7–39.7) on day 7 (*p* = 0.002) and 55.1% (IQR: 53.1–58.7) on day 10 (*p* = 0.0004) compared with the baseline (Table 1).

An analysis of the tumor cell number (TCN), defined as PLAP-positive cells, revealed a similar temporal pattern. No statistically significant change was observed between day 0 and day 3 (*p* = 0.18). However, a pronounced and statistically significant decrease in TCN was evident by day 7, with a median reduction of 27.5% (IQR: 23.7–33.0; *p* = 0.0033), which further progressed by day 10 to 53.2% (IQR: 51.2–56.8; *p* = 0.00018) relative to the control. The greater relative decline in TCN compared with OCN indicates the preferential loss of tumor cells and the comparatively higher short-term resilience of stromal/microenvironmental components under EVOC conditions.

The immunohistochemical assessment of the proliferative activity using Ki-67 demonstrated a progressive and decline in the proliferation index (PI) over time. A modest but significant decrease was observed already at day 3 compared with day 0 (*p* = 0.041), followed by a marked reduction at day 7 (*p* = 0.0024) and day 10 (*p* = 0.0002). Quantitative summary statistics for the OCN, TCN, stromal cell number (OCN-TCN), and PI are provided in Table 1.

### 2.5. EVOC Analysis

Based on the evaluation of the overall cell number (OCN), tumor cell number (TCN), and proliferative index (PI)—the proportion of positively stained cells in the reaction with antibodies to Ki-67—it was decided that PI was the most representative indicator, due to its more accurate reflection of the true proliferative potential of tumor cells. Therefore, for the analysis of the EVOC model, it was decided to consider the area under the curve (AUC) for PI as an integrative indicator of tumor viability (Figure 3).

## 3. Discussion

In this study, we adapted the ex vivo organ culture (EVOC) model for non-metastatic seminoma cT1–3, cN0–3 without distant metastases (M0), maintaining the viability of atypical cells for up to 10 days. During this period, we were able to preserve the viability of both tumor cells and cells of the tumor microenvironment.

The challenge of creating preclinical models that accurately reflect the in vivo tumor landscape is a central theme in oncology research. Traditional two-dimensional (2D) cell cultures, while foundational to cancer research, are increasingly seen as inadequate for fully recapitulating the complex in vivo environment of tumors. As noted by Kapałczyńska et al., 2D cultures can lead to an altered cell morphology, polarity, and gene expression, which may not accurately reflect the behavior of cancer cells within a patient [28]. This is a critical limitation, as the interactions between cancer cells and their surrounding microenvironment are known to play a crucial role in tumor progression, metastasis, and the response to therapy.

The development of three-dimensional (3D) culture models (most often described in the literature are precisely spheroids and organoids), has been a significant step forward in addressing the limitations of 2D systems. These models can more accurately mimic the cell–cell and cell–matrix interactions that are characteristic of solid tumors. However, as Jubelin et al. have pointed out, even these advanced 3D models have their own set of limitations: for example, the process of creating organoids often involves the dissociation of the original tumor tissue and the use of artificial matrices, which can alter the native architecture and cellular composition of the tumor microenvironment [14].

Within this evolving landscape of in vitro modeling, EVOC offers a complementary approach that maintains intact tumor fragments at an air–liquid interface, aiming to preserve not only tumor cells but also the key stromal and immune components of the tumor microenvironment (TME). While various in vitro and in vivo systems, as organoids, explants, and xenografts, have contributed substantially to modeling germ cell tumors, each approach retains inherent methodological constraints. In this context, our work should be viewed as a complementary, pilot attempt to explore the biological feasibility of maintaining the seminoma tissue integrity ex vivo using the EVOC framework. This study contributes to the broader methodological continuum aimed at improving the physiological relevance of tumor models.

Due to the above-mentioned properties, the EVOC model is practically indispensable when studying primarily the fundamental biological properties of a tumor. However, in addition to fundamental research, the EVOC model also has applied clinical significance. The application of this model to determine chemotherapeutic sensitivity has already shown significant concordance between the results obtained with EVOC and the actual clinical response to treatment. Thus, in the largest study to date of an EVOC application, conducted by Golan et al., a specificity of 77% and a sensitivity of 96% of this method were achieved [29]. Thus, the EVOC technique has already been successfully used on tumors of the bladder, lung, breast, head and neck, brain, esophagus, stomach, intestine, and pancreas [30,31,32]. In addition, at least one clinical trial on the adaptation of the EVOC model for metastatic tumors (NCT04599608) has been registered to date.

We believe that the EVOC model is an extremely useful analysis tool in the case of such unique malignant diseases as seminoma. The formation of germ cell tumors, particularly seminomas, is a complex, multi-stage process rooted in aberrant embryogenesis. This process initiates when damaging events lead to the arrest or abnormal differentiation of primordial gonocytes. This phase is often accompanied by the accumulation of genetic abnormalities, which, in conjunction with environmental factors, culminates in the malignant transformation of these cells [33,34].

A critical aspect of seminoma pathogenesis involves the proliferation of immature spermatogonia within the germinal epithelium of the seminiferous tubules. The most probable model suggests that arrested gonocyte maturation, coupled with the persistence of embryonic features and increased genomic instability, forms the basis for carcinoma in situ (CIS). The progression from CIS to invasive cancer is frequently associated with a gain on the short arm of chromosome 12, specifically an Isochromosome i (12p), which may involve genes such as KRAS2 and NANOG pseudogenes. Furthermore, seminomas often exhibit an increased frequency of isozymes of chromosomes 7, 15, 19, and X [35,36].

But the remarkable resistance of spermatogonial stem cells to mutational changes (often considered a cell population with one of the lowest spontaneous mutation rates and a robust capacity to maintain genetic integrity post-chemotherapy [37]), the phenomenon of a mutant primordial gonocyte appearance, remains a significant area of study. One compelling explanation for this paradox is the high hereditary predisposition to testicular germ cell tumors, with approximately half of the cumulative risk attributed to congenital genetic factors [38].

Seminomas typically present as a unilateral, firm-to-hard palpable mass within the testis. On gross examination, these tumors are characterized by pale grey to yellow nodules that are often uniform or slightly lobulated, frequently bulging from the cut surface. While generally homogeneous, larger masses may exhibit heterogeneity due to areas of hemorrhage and necrosis [39,40].

Histopathologically, seminomas are broadly categorized into classical, spermatocytic, and syncytiocytotrophoblastic types. The classical seminoma, accounting for approximately 85% of cases, is characterized by monotonous sheets of large cells with abundant clear cytoplasm, round hyperchromatic nuclei, and prominent nucleoli, with infrequent mitoses. Anaplastic seminomas, representing about 10% of cases, are defined by a high mitotic index (≥3 mitotic figures per high-power field), though this designation does not necessarily imply a more aggressive clinical course. The spermatocytic type, a rarer form (5%), typically affects older men (above 60 years) and is notable for its well-differentiated cells resembling secondary spermatids and a lower propensity for metastasis. The syncytiocytotrophoblastic subtype is associated with elevated serum βhCG levels [41,42].

A distinctive feature of seminomas is their macrophage-rich tumor microenvironment. The formation of this environment is partly driven by the active secretion of prostaglandins, which serve as potent chemoattractants for immune cells [43,44]. The functional orientation of these tumor-associated macrophages (TAMs) in seminomas is a subject of ongoing debate. Both pro-tumorigenic M2-like and anti-tumor M1-like phenotypes have been reported, and the dynamic balance between these populations is hypothesized to dictate whether immune infiltration promotes or constrains tumor growth [45].

This biological and molecular complexity of seminoma raises a fundamental question: how can such heterogeneity (encompassing genetic alterations, stromal interactions, and immune cell dynamics) be faithfully reproduced in an experimental setting? Current preclinical systems often fail to preserve this intricate tissue organization, which limits their predictive value for therapeutic testing. In this context, the EVOC model may also be useful in studying tumor biology specifically.

The fact is that, despite the high degree of curability among these pathologies, both in the case of seminomas and non-seminomatous germ cell tumors, the standard treatment strategy is accompanied by significant iatrogenic complications, as hypogonadism and male infertility [42]. Thus, 18–20% of patients with GCTs of stage IS-III have resistance or reduced sensitivity to platinum-based drugs, which negatively affects the prognosis of these patients. Therefore, targeted therapy has become one of the directions in the development of new treatment strategies for chemoresistant germ cell tumors. Clinical trials are already underway using checkpoint inhibitors: durvalumab, ipilimumab, and nivolumab [46,47]. And that is precisely why taking an additional look at the biological basis of the tumor and the microenvironment of seminoma and understanding the various processes involved can assist scientists.

Until now, adaptation of the EVOC model for testicular tumors has not been performed. In the course of the literature analysis, we found two studies in which a seminoma explant was a by-product, was not subjected to specialized analysis, and lasted 2 h and 2 days, respectively [12,48]. The closest to ours was an innovative study conducted by Danyang Wang et al., in which they managed to maintain the viability of the testicular tissue of infants with cryptorchidism for 60 days [49]. A separate issue of the clinical application of the EVOC model remains the search for standardized methods for assessing the degree of influence of chemotherapeutic, targeted drugs, radiation, or virotherapy on the viability of tumor cells, as well as an analysis of the results obtained with the further formation of the most optimal treatment strategy. Building upon these findings, we aimed to explore the feasibility of adapting the EVOC platform specifically for seminoma tissue.

Due to the pilot nature of the study, we encountered some difficulties in the process of selecting the necessary nutrient medium for culturing tumor cells. Thus, using the recommended RPMI (Roswell Park Memorial Institute 1640) medium supplemented with 10% fetal bovine serum [49], we were unable to maintain the proliferative activity of tumor cells for more than 5 days. Furthermore, within our laboratory, this medium proved to be more susceptible to contamination by exogenous microorganisms, despite the enrichment of the medium with broad-spectrum antibiotics.

The initial failure of RPMI 1640 medium to sustain seminoma viability beyond five days is not merely a technical issue, but a reflection of the distinct metabolic demands of this tumor type, which requires deeper contextualization than the previously speculative explanation involving dissociation enzymes. Seminomas, like many germ cell tumors, exhibit a pronounced Warburg effect, characterized by a high rate of aerobic glycolysis and a corresponding high demand for glucose [50,51]. A comparative analysis of the cultural media reveals that RPMI 1640 is a less nutrient-rich formulation, containing a relatively low glucose concentration (2.0 g/L) and lacking the pyruvate and higher amino acid concentrations found in DMEM/F12 [52]. The successful transition to the more universal DMEM/F12 medium, which possesses a significantly higher glucose concentration (4.5 g/L) and a broader spectrum of micronutrients, directly addresses the high glycolytic and biosynthetic demands of the seminoma tissue [53]. Therefore, the performance of DMEM/F12 is likely attributable to its ability to better support the high metabolic flux of the intact tumor tissue, which, unlike dispersed cell lines, cannot rapidly adapt its nutrient uptake mechanisms. This evidence-based comparison underscores the necessity of matching the culture medium’s nutritional profile to the known metabolic phenotype of the tumor in EVOC models.

In addition to optimizing the culture medium, the practical value of our work lies in creating controlled model for ex vivo organ cultivation that allows us to modulate the tumor microenvironment. One of the key parameters determining culture stability was the oxygen concentration. During pilot tests, we evaluated different oxygen levels in a three-gas incubator and confirmed that an atmospheric concentration of 20% O_2_ at 37 °C and 5% CO_2_ provides optimal preservation of the seminoma morphology and its proliferative potential. This finding is consistent with previously published data indicating that tissues preserved at physiological oxygen levels exhibit lower levels of oxidative stress and apoptosis compared to hyperoxic conditions [54]. The combination of a moderate oxygen concentration, stable temperature, and strictly controlled humidity proved to be critical for maintaining tissue viability for ten days.

Maintaining sterility throughout the process was equally important. All manipulations, including medium replacement, sampling, and collection, were performed in a Class II laminar flow cabinet using sterile instruments and consumables. This ensured the absence of microbial contamination, which was further confirmed by the absence of turbidity or odor in the culture medium during the observation period.

Another important methodological improvement was the introduction of a porous membrane substrate that prevented tissue fragments from sinking into the liquid phase and ensured effective gas exchange at the air–liquid interface. Comparative observations showed that in the absence of the insert, tumor fragments quickly lost viability, demonstrating an early decrease in the proliferation index and decay of the cytoarchitecture. In contrast, cultures supported on a porous scaffold showed stable Ki-67 expression and an intact tissue structure until day 10. These results confirm the well-known principle that the air–liquid interface provides adequate oxygenation from above, allowing nutrients to diffuse from the basal medium, thereby recreating a pseudo-vascular environment ex vivo [16].

Based on a histological examination during OCN and TCN counting for the seminoma EVOC model, it was found that the tumor cell population remains relatively stable by day 3 and decreases significantly by days 7–10, which is explained by the biological properties of the tumor cells. Atypical cells receive the necessary nutrients primarily through aerobic glycolysis, synthesizing all the structural molecules necessary for cell division by consuming glucose, for example, through the pentose phosphate pathway, and therefore quickly deplete its reserves in the nutrient medium [55]. In addition, it is known that the proliferation of seminoma cells is supported by endocrine and paracrine factors produced by Leydig and Sertoli cells, so the absence of hormonal stimulation in the culture medium also reduces their survival [19]. One of the differences between seminoma cells, compared to normal seminiferous tubule cells, is the reduced activity of autophagy processes, which are often necessary for adaptation to changing environmental conditions and under the influence of damaging factors [56].

This observation is important in the context of the testicular microenvironment, particularly the Sertoli and Leydig cells, which secretes a complex paracrine network of growth factors and hormones essential for germ cell homeostasis and proliferation [57,58]. Accordingly, the time-dependent decline in both the PI and TCN observed in the EVOC system is likely to reflect, at least in part, the dependence of seminoma cells on these microenvironment-derived cues. Rather than representing a nonspecific culture-related effect, this decline may therefore indicate reduced proliferative support following removal from the native testicular niche. Future refinements of the model may aim to address this limitation by incorporating defined Sertoli- or Leydig-derived factors into the culture medium or by developing co-culture approaches to partially reconstitute key paracrine interactions [59].

When counting microenvironment cells, calculated as (OCN-TCN), their slower rate of death was noticeable, which is explainable from the point of view of changes in their metabolism. In the cells of the tumor microenvironment and peritumoral zone, there is usually no adequate oxygenation, and therefore, the cells are adapted to stressful conditions, so they more successfully tolerate extraction from the primary tumor and seeding in the culture medium.

An analysis of the proliferative index, assessed as the proportion of Ki-67-positive tumor cells, revealed a decrease in PI as early as day 3 in the seminoma material. On days 7 and 10, the proliferative index also decreased, with the lowest value observed on day 10. Due to the greater sensitivity of the PI indicator, it was decided to use it as an integrative representative marker of tumor viability, calculated as the AUC for the PI curve.

The Ki-67 proliferative index observed in baseline seminoma samples approached 90%, which lies at the upper end of values reported in the literature [60,61,62]. Although Ki-67 indices exceeding 50% are commonly described in seminomas, values close to 90% are less frequently reported. This elevation is likely attributable to the methodological aspects of quantification, including a whole-slide digital analysis focused on tumor regions rather than selected high-power fields, as well as to the intrinsically high proliferative activity in the analyzed specimens. An independent manual review of representative sections confirmed widespread Ki-67 nuclear positivity among tumor cells at day 0. Importantly, Ki-67 expression in seminoma has not been shown to correlate robustly with the tumor stage or clinical aggressiveness, suggesting that the high baseline index reflects biological characteristics rather than adverse tumor behavior. Irrespective of absolute values, the central observation of the present study is the pronounced relative decline in Ki-67 positivity (from approximately 89% at baseline to 44% by day 10), indicating a substantial reduction in the proliferative fraction during ex vivo organ culture.

The rationale for using the AUC of the proliferative index is to capture the overall proliferative potential of the tissue throughout the culture period, rather than at a single endpoint. However, the EVOC score remains a preliminary metric. We acknowledge that we have not yet established its reproducibility or predictive value. The current study did not formally assess inter-sample variability in EVOC scores, and we did not correlate these scores with external benchmarks such as actual cell viability assays or clinical outcomes. Thus, while the EVOC score provides a convenient summary, it should be interpreted with caution. Its validity will need to be confirmed in future studies involving larger sample sizes and known positive controls (e.g., testing a cytotoxic drug where a meaningful EVOC score change should correlate with cell death). Potential limitations of the EVOC score include sensitivity to baseline proliferative differences between samples and to technical variability in Ki-67 quantification. Despite these caveats, we propose the EVOC score as a starting point for quantitatively comparing conditions in EVOC models, with the goal of refining it as more data become available.

However, we believe that it is important to consider that when analyzing the viability of tumor cells, it is necessary to consider not only the AUC value but also the shape of the graph itself. The fact is that in clinical practice, when analyzing malignant neoplasms of the testis, there are mixed variants of tumors consisting of components of seminoma, embryonal carcinoma, teratoma, choriocarcinoma, etc., which may have different biological properties and different chemotherapeutic sensitivity [63]. Moreover, even monomorphic germ cell or other solid tumors can possess different phenotypes from the point of view of genetic, epigenetic, and transcriptional activity, which explains the formation of chemotherapeutic sensitivity during treatment as a result of a microevolutionary process [64,65,66].

**Figure 4:**
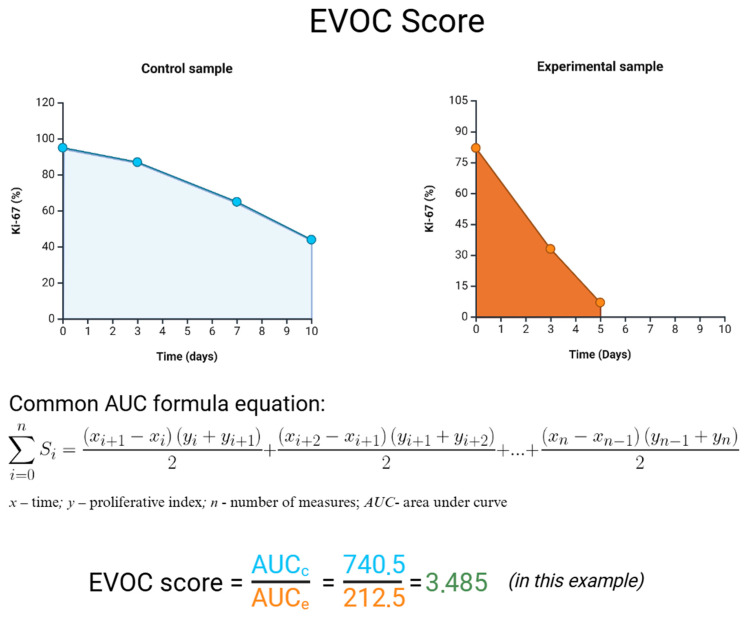
EVOC score calculation formula.

We believe that the most optimal option for standardizing the assessment of changes in the biological properties of a tumor depending on the nutrient medium, the introduction of growth factors, chemical, physical, or biological effects, and the possible response to chemotherapy, in addition to the visual assessment of the PI curve, will be the EVOC score, calculated as the ratio of the Ki-67 AUC of the control sample to the Ki-67 AUC of the experimental sample taken from the primary tumor. An example of calculating the EVOC score is presented in Figure 4, which shows a comparison of Ki-67 indicators for seminoma samples cultured in DMEM/F12 medium (control sample) and in RPMI medium (experimental sample), taken from the same primary tumor.

The successful selection of an anti-tumor culture medium necessary for maintaining viability, the preservation of a significant number of cellular elements of the microenvironment in the studied material for up to 10 days is a promising result, which indicates the successful adaptation of the EVOC model for non-metastatic seminoma of the testis and also points to a potentially successful analysis of the effectiveness of standard and targeted anti-tumor therapy aimed at both tumor cells and the tumor microenvironment.

Despite the successful adaptation of the EVOC model to seminoma tissue, several methodological aspects should be considered when interpreting the present findings. The study was designed as a pilot investigation and therefore involved a limited number of samples, which constrains the formal assessment of inter-sample variability, including variability of the EVOC score. The analyses focused primarily on morphometric and immunohistochemical parameters (TCN, PI, and histoarchitecture), which are appropriate for evaluating tissue viability and structural preservation but do not encompass functional readouts such as apoptosis, metabolic activity, or intracellular signaling. In addition, the present work was not intended to evaluate therapeutic responses, and no cytotoxic or targeted agents were applied, as the primary aim was to establish the feasibility and stability of the EVOC platform for seminoma. Finally, the culture conditions did not include exogenous hormonal supplementation, which may partially account for the gradual reduction in proliferative activity over time. Collectively, these considerations define the current study as a methodological and proof-of-concept framework that provides a basis for future studies incorporating larger cohorts, functional endpoints, and therapeutic interventions.

Taken together, these findings indicate that adapting the ex vivo organ culture (EVOC) platform to seminoma tissue is biologically relevant. Although the study may appear modest at first glance, it represents the first pilot implementation of this model in non-metastatic seminoma worldwide. By maintaining the native tumor cytoarchitecture and microenvironment, this approach provides a fundamentally novel method for understanding biology (proliferative step) in germ cell tumors. Further investigations should aim to refine this model and expand its application to other germ-cell tumor subtypes, ultimately strengthening its role as a bridge between experimental and clinical biology.

## 4. Material and Methods

### 4.1. Specimens

Tumor samples were taken from patients undergoing treatment in the Department of urology and chemotherapy of the P. Hertsen Oncology research Institute of the National medical research radiological centre (Moscow, Russia) institution with a diagnosis of a germ cell tumor (GCT), established at an oncological consultation based on the results of clinical, laboratory, and instrumental diagnostics.

All patients were scheduled for surgery in the volume of orchiectomy with adjuvant chemotherapy. The material was taken intraoperatively. All cases (n = 12) were histologically confirmed as non-metastatic seminoma pT1 and pT2 stages by a certified pathologist on the day 0 sample.

Inclusion and exclusion criteria are presented in Table 2.

### 4.2. Description of the Procedure and Intraoperative Sampling

**Figure 5:**
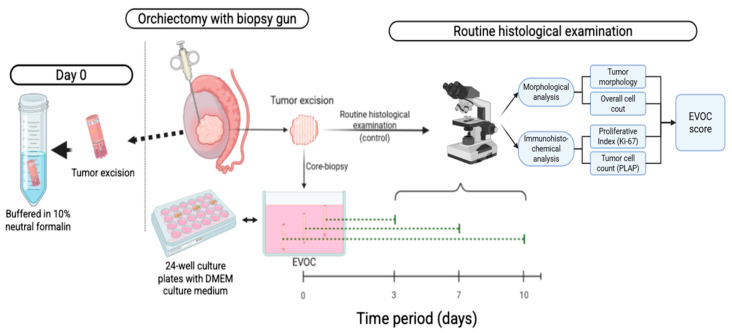
EVOC (ex vivo organ culture), steps of manipulations.

The schema of manipulation is illustrated in Figure 5. Intraoperatively, under sterile conditions during orchiectomy, tissue samples were taken using a reusable 14-gauge core biopsy gun, yielding cores approximately 1.5 mm in diameter and 15 mm in length. A portion of the core biopsies (seminoma material) was fixed in a neutral 10% buffered formalin solution for subsequent examination, which was used as a control (day 0). The «day 0 sample» served as the pre-culture control for both histological and immunohistochemical analyses and was processed and embedded in paraffin identically to the cultured samples to ensure direct comparability across all time points. Five to seven additional cores from spatially distinct fragments were allocated to ex vivo culture and harvested on days 3, 7, and 10 (one fragment per time-point).

Remaining cores were placed intraoperatively into pre-cooled DMEM/F-12 (Dulbecco’s Modified Eagle Medium/Nutrient Mixture F-12; Gibco, Thermo Fisher Scientific, Waltham, MA, USA) supplemented with antibiotics/antimycotics and transported to the laboratory in an insulated carrier with cold packs. DMEM/F-12 was selected as a broadly supportive basal medium for mammalian tissues; where indicated, a GlutaMAX^TM^-stabilized L-glutamine formulation (Thermo Fisher Scientific, Waltham, MA, USA) was used to reduce ammonia build-up.

Culture medium and supplements: DMEM/F12 culture medium supplemented with 10% fetal bovine serum, penicillin 100 IU/mL, streptomycin 100 μg/mL, amphotericin B 2.5 μg/mL, gentamicin sulfate 50 μg/mL, and L-glutamine 2 mM (292 µg/mL). The L-glutamine concentration was chosen to align with standard cell culture practices for optimal cell viability and proliferation.

### 4.3. Ex Vivo Organ Culture

Each sample for the study was obtained at 3 time points: on days 3, 7, and 10. These time points were selected based on previous studies demonstrating viable seminoma tissue culture up to 7 days [12] and pilot experiments in our laboratory indicating a preserved tissue architecture and proliferative activity up to 10 days.

All sample handling and manipulations were carried out under strict aseptic conditions within a class II laminar-flow biosafety cabinet (vertical airflow, minimum face velocity: 0.3–0.5 m/s), with all instruments and consumables sterilized (autoclaved or gas-sterilized) prior to use. Laboratory personnel wore sterile gloves, gowns, and surgical masks, and used sterile forceps and blades.

*Culture setup:* upon arrival in the laminar hood, tumor cores were gently rinsed in sterile phosphate-buffered saline (PBS, pH 7.4; Thermo Fisher Scientific, Waltham, MA, USA) and then transferred into pre-labeled, sterile 24-well culture plates. Each fragment was carefully placed onto a porous cell-culture insert so that it rested on the membrane and did not sink into the medium. Specifically, a 0.4 µm pore polycarbonate membrane insert (Merck KGaA, Darmstadt, Germany) was used. The insert was seated into a tissue-culture-treated well (Paneco Ltd., 24-well plate, Moscow, Russia). The culture medium (Section 4.2) was added beneath the insert until the basal surface of the insert contacted medium but without submerging the tissue (i.e., establishing air–liquid interface). All fluid additions and removals were performed slowly via pipette to avoid disrupting the tissue position, using sterile filter tips.

After setup, the plates were immediately transferred, in sealed sterile containers, into a tri-gas incubator (Heracell^TM^ VIOS 160i, Thermo Fisher Scientific, Waltham, MA, USA) maintained at 37 °C, 5% CO_2_, and 20% O_2_. The medium was exchanged every 24 h: under laminar flow, the spent medium was removed from the well (without touching the tissue), and fresh sterile pre-warmed medium was added to the same volume beneath the insert.

On days 3, 7, and 10, the culture plates were retrieved (inside a sealed container) and returned to the laminar hood. Under sterile conditions, the insert was gently lifted using sterile forceps; the tissue fragment was excised (if adhered) or carefully lifted with a sterile spatula and immediately immersed into 10% neutral-buffered formalin (pre-warmed to room temperature). Instruments used for dissection were sterilized or flame-sterilized between samples. The remainder of the well (if any tissue remained) was left in culture until the next time point. Each harvested tissue was then processed for paraffin embedding identically to the day 0 control.

### 4.4. EVOC Analysis

In seminoma samples, the following were evaluated: (a) histoarchitecture; (b) tumor cell number (TCN)—PLAP-positive cells; (c) proliferation index (PI)—the number of Ki-67-positive tumor cells; (d) total cell number (OCN). Next, for each of the above indicators, a curve graph was constructed, after which the area under the curve (AUC) was calculated using the trapezoidal rule. The AUC values for each patient’s sample were used as an aggregate measure of that parameter over the 10-day culture period, allowing for a quantitative comparison of overall changes in tissue viability and proliferation.

Cell number quantification was performed using QuPath image analysis software (Open Software for Bioimage Analysis, https://qupath.github.io, accessed on 12 September 2025) [67]. Whole-slide digital images were analyzed using QuPath (version 0.4.3).

To ensure a comprehensive and unbiased assessment while maintaining the necessary distinction between cell populations, a multi-step, reproducible protocol was implemented: the tumor region (seminoma) was manually delineated on the whole-slide image (WSI) by a board-certified pathologist to create a region of interest (ROI), thereby excluding adjacent stromal and non-neoplastic tissue from the subsequent analysis. Cell detection was performed within the defined tumor ROI using the «Positive Cell Detection» command. The parameters were specifically tuned for seminoma nuclei stained with Hematoxylin and DAB. The detection image was set to the Hematoxylin channel (a requested pixel size of 0.5 µm/pixel was used for all measurements; the detection radius was set to a minimum of 5 µm and a maximum of 10 µm, corresponding to the typical size range of seminoma nuclei; the nucleus splitting threshold was set to 0.15 to accurately separate clustered nuclei; and the cell expansion was set to 3 µm to define the surrounding cell boundary). Color deconvolution was applied using the standard H-DAB stain vector to separate the Hematoxylin (nuclear counterstain) and DAB (PLAP or Ki-67 positive stain) components. Positive cells (PLAP-positive and Ki-67-positive) were identified by applying a minimum DAB optical density (OD) threshold of 0.15 to the nucleus, which was empirically determined to optimally distinguish true positive staining from the background. Cells with a mean nuclear DAB OD value exceeding this threshold were classified as positive. The final output included the total number of cells (OCN) and the number of positive cells (TCN for PLAP, PI for Ki-67) within the tumor ROI, allowing for the calculation of the proliferation index and tumor cell density.

### 4.5. Histochemical Examination

Testicular tumor tissue fragments were fixed in buffered formalin solution, processed automatically, embedded in paraffin blocks, and sectioned into serial slices with a thickness of 2 µm. The sections were then deparaffinized, dehydrated, and stained with Hematoxylin and eosin and Periodic acid–Schiff (PAS). The histological specimens were examined under a Leica DM3000 microscope with microphotography (Leica Microsystems, Wetzlar, Germany). The evaluation of the specimens was conducted according to standard histological criteria.

The histoarchitecture was evaluated by a board-certified pathologist, focusing on the preservation of typical seminoma histological features, including uniform sheets of tumor cells with lymphocytic stroma, and the absence of extensive necrosis or degenerative changes. PAS staining was utilized to confirm the presence of glycogen within seminoma cells, a characteristic feature of this tumor type [68].

### 4.6. Immunohistochemical Examination

For the immunohistochemical analysis, the following primary antibodies were used: monoclonal antibodies against Ki-67 (Clone MM1, ThermoFisher, Waltham, MA, USA) and PLAP (placental alkaline phosphatase, a marker of seminoma cells; clone NB10 Cell Marque, Rocklin, CA, USA). For secondary antibody detection, a universal two-component HiDef Detection™ HRP Polymer system (Cell Marque, Rocklin, CA, USA) was used, including anti-IgG mouse/rabbit antibodies, horseradish peroxidase (HRP; Thermo Fisher, Waltham, MA, USA), and the DAB (3,3′-diaminobenzidine; Thermo Fisher, Waltham, MA, USA) substrate. Cell nuclei were counterstained with Mayer’s Hematoxylin. Antibody dilutions and incubation conditions followed the manufacturer’s standard protocols for automated IHC staining. After DAB substrate application, slides were counterstained with Mayer’s Hematoxylin.

The proliferation index (PI) refers to the percentage of Ki-67-positive tumor nuclei among total tumor nuclei in 10 fields of view at a magnification of ×400.

To validate the digital quantification of the Ki-67 proliferative index, two independent pathologists visually assessed Ki-67 staining on a subset of day 0 slides. In randomly selected fields, the manually estimated Ki-67-positive fraction of tumor cells (approximately 85–90%) closely matched the QuPath-derived values, confirming the accuracy of the automated method. Ki-67 was positivity defined, using a high DAB optical density threshold to count only for strongly stained nuclei, minimizing false-positives.

### 4.7. Statistical Analysis

Statistical analysis was performed using STATISTICA 13.5.0.17 (TIBCO Software Inc., Palo Alto, CA, USA). Continuous variables were summarized as the mean ± standard deviation (SD) when approximately normally distributed and as the median with the interquartile range (IQR) otherwise. The normality of distributions was assessed using the Shapiro–Wilk test complemented by the visual inspection of histograms and Q-Q plots, performed separately for each outcome variable at each time point. Primary outcome measures included the overall cell number (OCN), tumor cell number (TCN), stromal/microenvironment cell number (OCN-TCN), and proliferation index (PI). Temporal changes across culture time points (day 0, 3, 7, and 10) within the same patient-derived samples were analyzed using one-way repeated-measures analysis of variance (ANOVA). Sphericity was assessed using Mauchly’s test; when the sphericity assumption was violated, Huynh–Feldt correction was applied, and corrected degrees of freedom were reported. When a significant main effect of time was detected, post hoc pairwise comparisons between time points were conducted with an appropriate adjustment for multiple testing (Tukey’s honestly significant difference test or Bonferroni-adjusted paired comparisons, as applicable). Effect sizes were calculated to quantify the magnitude of observed changes. The partial eta-squared (ηp^2^) was reported for repeated-measures ANOVA, and Cohen’s dz was used for paired comparisons. Where relevant, 95% confidence intervals (CIs) for mean differences were provided. Given the limited sample size, non-parametric Friedman tests were additionally performed as sensitivity analyses when distributional assumptions were borderline; these analyses did not alter the overall interpretation of results. Categorical variables were expressed as frequencies and percentages and compared using chi-square or Fisher’s exact test, as appropriate. All statistical tests were two-tailed, and a *p*-value < 0.05 was considered statistically significant.

## 5. Conclusions

This study presents the first-time pilot study of the ex vivo organ culture (EVOC) model for non-metastatic seminoma, enabling the prolonged maintenance of tumor and microenvironmental cell viability for up to 10 days. We established optimal culture conditions and introduced an integrative approach to evaluate proliferative activity under various experimental influences.

## Figures and Tables

**Table 1:** Quantitative morphometric analysis of seminoma EVOC over time.

Day	0	3	7	10
**OCN** (cells)	47,595.2 ± 3617.6 (95% CI: 4529.5–4989.5)	42,166.7 ± 3494.2 (95% CI: 39,980.0–44,353.4)	32,109.4 ± 3649.9 (95% CI: 29,720.1–3449.9)	21,413.17 ± 1933.9 (95% CI: 20.186–22.640)
**TCN** (PLAP + cells)	40,611.89 ± 1890.5 (95% CI: 38,142.1–43,082.6)	37,960.3 ± 2811.3 (95% CI: 36,178.8–3974.9)	29,408.2 ± 3211.4 (95% CI: 27,303.6–3151.3)	18,986.72 ± 1197.4 (95% CI: 18.223–19.751)
**OCN-TCN** (stromal cells)	5983.3 ± 3685.2 (95% CI: 4642.1–9325.0)	4206.4 ± 2346.2 (95% CI: 2722.8–5691.2)	2695.17 ± 1059.9 (95% CI: 2026.0–3364.1)	2426.44 ± 1644.8 (95% CI: 1.372–3.481)
**PI** (Ki-67 + cells, %)	8947.0 ± 552.2 (95% CI: 8596.1–929.4)	8805.0 ± 642.4 (95% CI: 8398.0–921.5)	6800.2 ± 701.3 (95% CI: 6355.0–724.5)	4388.6 ± 550.1 (95% CI: 4038.3–473.8)

Note: all data are presented as the mean ± standard deviation (M ± SD).

**Table 2:** Inclusion and exclusion criteria.

Inclusion Criteria	Exclusion Criteria
Male sex Age from 18 to 65 years Primarily operable germ cell tumor with cT1–2, cN0–3 without distant metastases (M0) Clinically established diagnosis of malignant neoplasm of the testis Absence of prior drug and/or radiation therapy Functional status on the ECOG scale 0–1	Primary multiple cancer (synchronous or metachronous). Severe non-surgical comorbidity or acute infection (sepsis, uncontrolled arterial hypertension, uncontrolled diabetes mellitus, acute cerebrovascular accident, or myocardial infarction less than 6 months old, mental disorders, etc.). Hepatitis B or C, HIV. History of cryptorchidism or presence at the time of admission to the medical facility.

Note: ECOG—Eastern Cooperative Oncology Group performance status; Node, Metastasis classification (cT1–2: primary tumor stage; cN0–3: regional nodes; M0: no distant metastasis).

## Data Availability

The original contributions presented in this study are included in the article. Further inquiries can be directed to the corresponding authors.
